# Immunologische Aspekte nach der Lungentransplantation

**DOI:** 10.1055/a-2590-9933

**Published:** 2025-05-13

**Authors:** Caroline Hillebrand, Alberto Benazzo

**Affiliations:** 1 27271 Thoraxchirurgie, Medizinische Universität Wien, Wien, Österreich

**Keywords:** Transplantation, Lunge, Immunsuppression, transplantation, lung, immune suppression

## Abstract

Die Lungentransplantation hat sich seit den 1980er-Jahren durch Fortschritte in chirurgischen Techniken und der Einführung von Immunsuppressiva wie Ciclosporin zu einem etablierten Therapieverfahren entwickelt. Trotz verbesserter kurzzeitiger Ergebnisse bleibt die Langzeitprognose, insbesondere durch immunologische Komplikationen, eingeschränkt. Mit einer medianen Überlebenszeit von etwa 6 Jahren ist die Lunge aufgrund ihrer Exposition gegenüber Umweltantigenen und einer großen vaskulären Endothelfläche das solide Organ mit der größten Immunogenität. Nach einer Lungentransplantation spielen verschiedene Formen der Alloreaktivität, einschl. der T-Zell-vermittelten akuten und chronischen Abstoßungen eine zentrale Rolle. Ergänzend tragen humorale Immunantworten durch die Bildung donorspezifischer und Non-HLA-Antikörper zur Transplantatschädigung bei. Wiederkehrende Gewebeschäden, etwa durch Ischämie-Reperfusions-Schäden, führen zur Freilegung kryptischer Antigene, fördern autoreaktive Prozesse und begünstigen die Bildung tertiärer lymphatischer Organe. Diese Prozesse unterhalten eine anhaltende Entzündungsreaktion, die zur chronischen Transplantatdysfunktion führen kann. Abstoßungsreaktionen bleiben eine große Herausforderung. Akute Formen, wie zelluläre und humorale Abstoßungen, erfordern schnelle, gezielte Therapien, um irreversible Schäden zu verhindern. Chronische Abstoßungen, insbesondere die chronische Lungenallograft-Dysfunktion (CLAD) verschlechtern die Lungenfunktion langfristig. Die Unterscheidung der Hauptphänotypen der CLAD, dem Bronchiolitis-obliterans-Syndrom und dem restriktiven Allograft-Syndrom, ist entscheidend für Prognose und Therapie. Dennoch sind die Behandlungsmöglichkeiten limitiert, sodass eine Retransplantation oft die letzte Option darstellt. Die immunsuppressive Therapie bildet die Grundlage zur Prävention von Abstoßungen. Sie umfasst meist eine Dreifachkombination aus Calcineurininhibitoren, antiproliferativen Substanzen und Kortikosteroiden. Für die Induktionstherapie kommen monoklonale oder polyklonale Antikörper zum Einsatz. Moderne Strategien zielen darauf ab, Immunantworten effektiv zu unterdrücken und gleichzeitig schwerwiegende Nebenwirkungen wie Infektionen, Malignome und Nephrotoxizität zu minimieren. Zukünftige Forschungsansätze fokussieren sich auf personalisierte Immunsuppressionsstrategien, optimierte Diagnostik und innovative Therapieansätze, um die Langzeitprognose von Lungentransplantierten zu verbessern.

## Abstract (en)

Since the 1980 s, lung transplantation has evolved into an established therapeutic procedure, due to advancements in surgical techniques and the introduction of immunosuppressants such as cyclosporine. Despite improved short-term outcomes, the long-term prognosis remains limited, primarily due to immunological complications. With a median survival of approximately six years, the lung is the most immunogenic solid organ, owing to its constant exposure to environmental antigens and its extensive vascular endothelial surface. After lung transplantation, various forms of alloreactivity, including T cell-mediated acute and chronic rejection, play a central role. Additionally, humoral immune responses, characterised by the production of donor-specific and non-HLA antibodies, contribute significantly to graft injury. Recurrent tissue damage, such as ischemia reperfusion injury, leads to the exposure of cryptic antigens, promotes autoreactive processes, and facilitates the formation of tertiary lymphoid organs. These mechanisms sustain persistent inflammation, ultimately resulting in chronic graft dysfunction. Rejection reactions remain a major challenge. Acute forms, such as cellular and humoral rejection, require rapid and targeted therapies to prevent irreversible damage. Chronic rejection, particularly chronic lung allograft dysfunction (CLAD), progressively impairs lung function. In the main phenotypes of CLAD, bronchiolitis obliterans syndrome (BOS) and restrictive allograft syndrome (RAS), are crucial for prognosis and treatment. Nevertheless, therapeutic options remain limited, and retransplantation is often the last resort. Immunosuppressive therapy forms the cornerstone of rejection prevention, and typically employs a triple combination of calcineurin inhibitors, antiproliferative agents, and corticosteroids. Induction therapy frequently involves monoclonal or polyclonal antibodies. Modern strategies aim to effectively suppress immune responses while minimising severe side effects, such as infections, malignancies, and nephrotoxicity. Future research will focus on personalised immunosuppressive strategies, optimised diagnostics, and innovative therapies to improve the long-term prognosis of lung transplant recipients.

## Einleitung

Die erste Lungentransplantation beim Menschen wurde 1963 durchgeführt, doch trotz technischer Erfolge waren die Ergebnisse lange von hoher Mortalität geprägt 1 . Erst mit der Einführung von Ciclosporin und der Weiterentwicklung chirurgischer Techniken in den 1980er-Jahren gelang es, die Lungentransplantation zu einem klinisch etablierten Verfahren zu entwickeln 2 .

Die Immunologie der Lungentransplantation ist jedoch beispiellos komplex. Die einzigartige Immunogenität des Lungengewebes, bedingt durch die hohe immunologische Aktivität des pulmonalen Endothels und die permanente Exposition gegenüber der Umwelt, macht die Lunge zu einem der komplexesten und herausforderndsten Organe in der Transplantationsmedizin. Die permanente Immunstimulation aktiviert sowohl angeborene als auch erworbene Immunmechanismen, die nicht nur akute Abstoßungsreaktionen, sondern auch langfristige Komplikationen fördern. Bereits früh nach der Transplantation stellen Infektionen und Transplantatversagen aufgrund der primären Graftdysfunktion die Hauptursache für Todesfälle dar. Im weiteren Verlauf dominiert jedoch die chronische Abstoßung (chronische Lungenallograft-Dysfunktion, CLAD), die für etwa 40–50% der Todesfälle ab dem 1. Jahr nach der Transplantation verantwortlich ist 3 .

## Immunologische Grundlagen der Lungentransplantation

Das menschliche Immunsystem ist ein hochentwickeltes, dynamisches Netzwerk, das im Laufe der Evolution durch die ständige Interaktion mit mikrobiellen Pathogenen geformt wurde. Es übernimmt eine zentrale Rolle bei der Abwehr von Fremdantigenen und damit auch bei der Abstoßung und Akzeptanz von Transplantaten. Insbesondere die Lungentransplantation stellt aufgrund der kontinuierlichen Exposition des Organs gegenüber Umweltantigenen und Pathogenen eine besondere immunologische Herausforderung dar. Dabei spielen Prozesse wie die Erkennung fremder Antigene (Alloreaktivität) und die Fehlregulation körpereigener Abwehrmechanismen (Autoreaktivität) eine entscheidende Rolle.

### Alloreaktivität

Alloreaktivität beschreibt die Immunantwort des Empfängers auf fremde Antigene des Transplantats (Alloantigene), insbesondere auf MHC-Moleküle (Major Histocompatibility Complex), die auf den Spenderzellen exprimiert werden. Diese Reaktion wird durch die adaptive Immunität in einem T-Zell-vermittelten Prozess gesteuert, der zunächst die Antigenerkennung und anschließend eine proinflammatorische Reaktion umfasst, die zu einer Abstoßung führt. Im Zentrum der Alloreaktivität stehen 3 verschiedene Wege: der direkte, der indirekte und der semidirekte Weg 4 .

Der direkte Weg der Alloreaktivität beschreibt, wie antigenpräsentierende Zellen (APCs) des Spenders, sog. „Passenger Leukocytes“, in die sekundären lymphatischen Organe des Empfängers migrieren und dort naive T-Zellen aktivieren. Diese Aktivierung erfolgt durch die Erkennung allogener MHC-Moleküle auf den APCs des Spenders und führt typischerweise zu einer schnellen und akuten zellulären Reaktion, die zu einer Abstoßung des Transplantats führt. Der direkte T-Zell-Alloreaktionsprozess ist polyklonal und umfasst bis zu 10% des T-Zell-Repertoires 4 5 .

Der indirekte Weg der Alloreaktivität wurde erstmals in den 1980er-Jahren beschrieben 6 , als gezeigt wurde, dass Transplantate ohne Passenger Leukocytes dennoch abgestoßen werden können, was auf einen konventionellen Mechanismus der Antigenpräsentation hinweist. Die APCs des Empfängers präsentieren dabei MHC-Moleküle des Spenders auf ihren eigenen MHC-Klasse-II-Molekülen, wodurch CD4+ T-Zellen in den sekundären lymphatischen Organen aktiviert werden 7 . Anfangs zeigte die indirekte alloreaktive Reaktion ein oligoklonales Muster mit einer begrenzten Anzahl von T-Zell-Klonen, die auf wenige dominante Polymorphismen reagierten 8 . Im Verlauf breitet sich die T-Zell-Antwort jedoch auf zusätzliche nicht eigene MHC-Peptide aus, die zuvor unter der Aktivierungsschwelle blieben 9 . Dieser Prozess wird als Antigen-Spreading bezeichnet und ist ein zentraler Mechanismus bei der chronischen Abstoßung 10 . Die genauen Mechanismen, wie Empfänger-APCs Spenderantigene aufnehmen und verarbeiten, sind noch nicht vollständig geklärt. Vermutlich erfolgt die Aufnahme durch Pinozytose oder Phagozytose von abgestorbenen oder geschädigten Spenderzellen. Zudem spielen extrazelluläre Vesikel eine wichtige Rolle, da sie nicht nur Spenderantigene, sondern auch costimulatorische Moleküle transportieren, die für eine T-Zell-Aktivierung notwendig sind. Traditionell wird der indirekte Weg hauptsächlich mit chronischer Abstoßung in Verbindung gebracht 11 .

Der semidirekte Weg der Alloreaktivität basiert auf der Übertragung intakter Antigene zwischen verschiedenen Leukozyten, z. B. durch Trogozytose oder die Freisetzung extrazellulärer Vesikel 12 13 . Dies ermöglicht Empfänger-APCs, fremde MHC-Moleküle von Spenderzellen zu übernehmen, ein Prozess, der als MHC-Cross-Dressing bezeichnet wird 14 . Empfänger-APCs können Spender-MHC von Exosomen in den sekundären lymphatischen Organen aufnehmen und so eine T-Zell-vermittelte allogene Reaktion aktivieren. Ein Großteil der Exosomen exprimiert MHC-Klasse-II-Moleküle, die von Spender-APCs stammen. Zusätzlich werden nicht nur unverarbeitete Spenderantigene, sondern auch costimulatorische Signale wie IL-1β, FAS-Ligand, TRAIL und CD40 über die Exosomen übertragen. Diese Signale fördern die Aktivierung von Empfänger-APCs und tragen zur allogenen Immunantwort bei 15 .

**Allogene humorale Immunantwort**

In den letzten 2 Jahrzehnten wurde neben der T-Zell-vermittelten allogenen Antwort auch vermehrt Aufmerksamkeit auf die humorale Antwort gegen das Transplantat gerichtet, da B-Zellen eine zentrale Rolle bei der Transplantatschädigung spielen und die Immunantwort langfristig aufrechterhalten können. Den B-Zellen werden 5 Hauptfunktionen zugeschrieben: 1. Differenzierung in kurz- und langlebige antikörperproduzierende Plasmazellen (PC), 2. langfristige humorale Immungedächtnisfunktion, 3. Antigenpräsentation, 4. Bildung tertiärer lymphoider Organe und 5. Sekretion pro- und antiinflammatorischer Zytokine.

Die B-Zell-Entwicklung beginnt in den primären lymphatischen Organen und wird in den sekundären lymphatischen Organen abgeschlossen. Funktionelle B-Zell-Rezeptoren (BCR) entstehen durch DNA-Rekombination von V-, D- und J-Genabschnitten und ermöglichen die Erkennung vielfältiger Antigene. Nach der Selektion autoreaktiver B-Zellen verlassen Übergangs- und Marginalzonen-B-Zellen das Knochenmark, um ihre Reifung in der peripheren Zirkulation und in sekundären lymphatischen Organen abzuschließen. Chemokine lenken unreife B-Zellen in sekundäre lymphatische Organe, wo sie in Follikeln der Kortexregion organisiert sind.

B-Zellen werden aktiviert, wenn ihr BCR ein Antigen bindet. Entscheidend sind Signale durch den BCR und weitere Rezeptoren (z. B. CR2, FcγRII), die die Migration zur T-B-Zell-Grenze einleiten 16 . Dort interagieren sie mit CD4+ T-Helferzellen (Tfh), was zur Differenzierung in kurzlebige Plasmazellen oder Keimzentrums-(GC)-B-Zellen führt. Plasmazellen produzieren IgM-Antikörper, während GC-B-Zellen im Keimzentrum Affinitätsreifung, Klassenwechsel und somatische Hypermutation durchlaufen, gesteuert durch den Transkriptionsfaktor Bcl6. Nach der Expression eines funktionellen BCRs wandern GC-B-Zellen in die helle Zone und beginnen dort mit dem Antigenscreening auf follikulären dendritischen Zellen (FDC). Diese Interaktion wird durch Moleküle wie FcγR, CR2, CD40L auf den FDC sowie BCR, CD40 und IL-21R auf den GC-B-Zellen vermittelt. Nach erfolgreicher Selektion eines funktionellen BCRs treffen die GC-B-Zellen auf Tfh, was die weitere Reifung ermöglicht. Die Bindung des Peptid-MHC-Klasse-II-Komplexes, CD40 sowie CD80/86 auf der Oberfläche von B-Zellen an den TCR, CD40L und CD28 auf T-Zellen in Anwesenheit von IL-2, IL-4, IL-5 und IL-21 ist entscheidend für die erfolgreiche Reifung einer funktionsfähigen B-Zelle. Zusätzlich fördern ICOS:ICOSL- und SLAM-Familien-Interaktionen die Kommunikation zwischen Tfh- und B-Zellen. Das Schicksal der GC-B-Zellen wird durch diese Interaktionen bestimmt. Entweder kehren sie zur weiteren Diversifizierung in die dunkle Zone zurück oder sie differenzieren sich zu langlebigen Plasmazellen, gesteuert durch den Transkriptionsfaktor Blimp-1, oder zu Gedächtnis-B-Zellen. Plasmazellen wandern ins Knochenmark, während Gedächtnis-B-Zellen in sekundären lymphatischen Organen und der Peripherie zirkulieren 17 .

Die enge Zusammenarbeit zwischen T- und B-Zellen ist zentral für die allogene Immunantwort, da etwa das Fehlen oder die Blockade von T-Zellen die Produktion donorspezifischer Antikörper hemmt 18 19 .

**Antikörpervermittelte Schäden**

Donorspezifische Antikörper (DSA) gegen das HLA-System können entweder bereits vor der Transplantation durch Sensibilisierung (z. B. Schwangerschaft, Autoimmunerkrankungen, Transfusionen) vorhanden sein oder sich de novo nach der Transplantation entwickeln. Die Sensibilisierung von B-Zellen gegen nicht übereinstimmendes HLA erfolgt über die Aktivierung von CD4+ T-Zellen und APC durch den indirekten oder semidirekten Weg. Die Bindung von DSA an HLA-Moleküle auf Endothelzellen führt zu einer Aktivierung verschiedener Effektormechanismen: 1. Die IgG-Bindung aktiviert die Komplementkaskade, was zur Bildung des Membranangriffkomplexes (MAC) und zur Freisetzung von Chemokinen führt. Diese rekrutieren Monozyten, NK-Zellen, Neutrophile und T-Zellen, die über Zytokine wie IFN-γ, TNF-α und GM-CSF Gewebeschäden verursachen; 2. HLA-Antikörper aktivieren Signalkaskaden (z. B. FAK, PI3K, mTOR), die Proliferation und Aktivierung von Endothel- und glatten Muskelzellen fördern. Zudem wird die Sekretion von Von-Willebrand-Faktor und P-Selectin induziert, was Thrombozytenaktivierung und Entzündungen begünstigt; 3. Chemokine wie CCL3 und CCL4 rekrutieren weitere Immunzellen, während IFN-γ und TNF-α die Produktion von CXCL9 und CXCL10 anregen, die NK-Zellen anziehen und eine Rückkopplungsschleife auslösen, die die Immunantwort weiter verstärkt; und 4. Granzyme und Perforine, die von NK-Zellen freigesetzt werden, verursachen durch antikörperabhängige zellvermittelte Toxizität (ADCC) direkte Schäden an Endothelzellen.

Schließlich führt die Bindung von IgG an zirkulierende Antigene oder antigentragende Parenchymzellen zur Aktivierung von APCs und einer verstärkten T-Zell-Antwort, was den Gewebeschaden weiter vorantreibt 20 .

### Autoreaktivität

Autoreaktivität beschreibt die fehlgeleitete Immunantwort des Körpers, bei der körpereigene Strukturen als fremd erkannt und angegriffen werden. Dies kann durch die Aktivierung von T- und B-Zellen ausgelöst werden, die gegen Autoantigene gerichtet sind. Grundlegende Studien haben die zentrale Rolle von Autoantigenen (z. B. Kollagene, Haushaltsproteine) in der Entstehung von Non-HLA-Antikörpern nachgewiesen, die gegen Transplantate gerichtet sind. Insbesondere bei der Lungentransplantation wurden Kollagen V und K1-α-Tubulin als Hauptantigene identifiziert, die Autoreaktivität auslösen können.

Non-HLA-Antikörper lassen sich in Autoantikörper (gegen körpereigene Antigene) und Alloantikörper (gegen polymorphe Non-HLA-Antigene von Spendern) unterteilen 21 22 . Der erste Bericht über pathogene Autoantikörper stammt von Brasile et al., in dem Anti-Endothelzell-Antikörper (AECA) eine hyperakute Abstoßung eines HLA-kompatiblen Nierentransplantats verursachten 23 . Interessanterweise entwickeln manche Patient*innen Autoantikörper ohne Abstoßung oder Anzeichen einer Transplantatdysfunktion. Es wird daher vermutet, dass zusätzliche Faktoren, wie Ischämie-Reperfusions-Schäden (IRI), Entzündungen oder spezifische Antigenexpression die Immunantwort beeinflussen. Die Autoantigene variieren zwischen Organen. Am intensivsten untersucht wurde der Angiotensin-Typ-1-Rezeptor (AT1R). In mehreren Studien korrelierten präformierte oder De-novo-AT1R-Antikörper mit einer verschlechterten Nieren- und Herztransplantatfunktion. Transplantatbiopsien dieser Kohorte zeigten nur minimale C4d-Ablagerung, was auf die Aktivierung komplementunabhängiger Mechanismen hinweist 24 25 26 . AT1R-Antikörper binden an Endothel- und Muskelzellen, aktivieren ERK-Kinase, NF-κB und AP-1, ähnlich wie Angiotensin II. Biopsien wiesen zudem eine Expression von Gewebefaktor auf Endothelzellen und ein mikrothrombotisches Muster im Transplantat auf. In der Lungentransplantation wurden Kollagen V und K1-α-Tubulin als 2 Hauptantigene identifiziert, die eine autoimmune Schädigung des Transplantats auslösen können. Kollagen-V-Antikörper korrelierten mit akuten Abstoßungen und CLAD 27 28 29 . Kollagen V ist ein kryptisches Antigen, das unterhalb der Epithelzellen der Atemwege liegt und nach IRI oder immunvermittelter Schädigung den Immunzellen des Empfängers präsentiert wird 27 30 . K1-α-Tubulin ist ein Gap-Junction-Protein, das unter normalen Bedingungen nicht exponiert ist. Infolge von Gewebeschädigungen und -umbau, die während einer Abstoßung oder Ischämie auftreten, können diese Antigene jedoch freigelegt werden und sich somit in Zielstrukturen für Antikörperablagerungen und zellvermittelte Immunantworten verwandeln 29 .

Die aktuelle Hypothese besagt, dass IRI, alloimmune Reaktionen und chronische Entzündungen Endothel- und Epithelzellen schädigen. Dabei werden kryptische Antigene und intrazelluläre Proteine, die auf apoptotischen Vesikeln exprimiert sind, den APCs zugänglich. Autoreaktive T- und B-Zellen können sich in tertiären lymphoiden Organen im Transplantat vermehren und aktivieren, was Autoimmunantworten auslöst 31 . Ischämie-Reperfusions-Schäden, die alloimmune Antwort sowie die Ausbildung tertiärer lymphatischer Organe wirken wechselseitig, wobei z. B. IRI auch ohne Alloreaktivität eine Autoimmunreaktion induzieren kann 30 . Kryptische Antigene, die während IRI freigelegt werden, können rekrutierten autoreaktiven T-Zellen präsentiert werden 32 . Das Komplementprotein C3 fördert die Opsonierung von Selbstantigenen und stimuliert B-Zellen 33 34 . Die Alloreaktivität kann durch sog. „Epitope Spreading“ die Autoimmunantwort erweitern, indem neue Antigene präsentiert werden 35 36 . Zudem ermöglicht der indirekte alloimmune Signalweg durch molekulare Mimikry die Entwicklung autoreaktiver T- und B-Zellen 37 . Th17-Zellen, die durch IL-23 aktiviert werden, fördern chronische Entzündungen und Autoimmunität. Studien belegen die Rolle der IL-17-Achse bei der Abstoßung solider Organtransplantate, insbesondere bei der Immunreaktion auf Kollagen V in Lungentransplantaten 27 . In Tiermodellen führte die Th17-vermittelte Reaktion auf Kollagen V zu vaskulären Entzündungen und BOS-Läsionen, gesteuert durch IL-17 und IL-23. Eine Blockade von IL-17 schützte Allotransplantate vor BOS 38 39 40 . Th17-Zellen rekrutieren Neutrophile, was durch IL-17-induzierte Expression von CXCL8, CXCL1 und GM-CSF in Atemwegszellen und Fibroblasten vermittelt wird 41 42 43 . Sie fördern außerdem die lymphatische Neogenese und bilden tertiäre lymphatische Organe, in denen B-Zellen Keimzentren entwickeln 44 45 . IL-17 und IL-21 stimulieren APCs und fördern die Bildung dieser Organe durch CXCL13- und CCL19-Expression 46 47 . Eine andauernde Produktion von BAFF und die Expression von IL‑21 können bewirken, dass autoreaktive B‑Zellen überleben und sich vermehren 48 .

Diese Erkenntnisse vereinen die zentralen Mechanismen der Immunreaktion im Lungenallograft und erklären die Abfolge der Ereignisse, die zur chronischen Abstoßung führen. Zu Beginn verursacht die IRI Gewebeschäden, wodurch kryptische Antigene freigelegt, IL-17-produzierende Zellen aktiviert und Immunzellen rekrutiert werden. Spender- und Empfänger-APCs sowie CD4+ T-Zellen wandern in sekundäre lymphatische Organe und induzieren eine alloreaktive Antwort, die weitere Schäden am Lungenallograft verursacht. Chronische Entzündungsreize verhindern die Heilung und fördern eine persistierende Präsentation von Allo- und Autoantigenen, wodurch IL-17-sezernierende Zellen dauerhaft aktiviert werden. Tertiäre lymphatische Organe und Keimzentrumsreaktionen intensivieren die Immunantwort, indem sie funktionsfähige Antikörper produzieren. Schließlich fördern Fibroblasten und Epithelzellen die Umgestaltung der extrazellulären Matrix und den epithelial-mesenchymalen Übergang, was in einer fortschreitenden Fibrose des Transplantats mündet.

### Besonderheiten der Lunge als transplantiertes Organ

Die Lunge weist im Vergleich zu anderen transplantierten Organen mehrere immunologische Besonderheiten auf, die ihre hohe Immunogenität erklären. Als Barriereorgan steht sie in direktem und konstantem Kontakt mit der äußeren Umwelt und ist kontinuierlich einer Vielzahl von Antigenen und Pathogenen ausgesetzt. Diese permanente Stimulation aktiviert das angeborene Immunsystem über Signalwege wie Toll-like-Rezeptoren (TLRs) und fördert dadurch sowohl angeborene als auch adaptive Immunmechanismen.

Ein weiterer entscheidender Faktor ist die große vaskuläre Endothelfläche der Lunge, die sie besonders anfällig für IRI macht. Solche Schäden aktivieren das Komplementsystem und führen zu einer vermehrten Rekrutierung von neutrophilen Granulozyten und Makrophagen. Die daraus resultierende entzündliche Mikroumgebung fördert eine frühzeitige T-Zell-Aktivierung und beschleunigt so die Abstoßung des Transplantats.

Darüber hinaus zeichnet sich die Lunge durch eine hohe Dichte an lymphatischem Gewebe und geweberesidenten Immunzellen aus. Dazu zählen mononukleäre Phagozyten und geweberesidente Gedächtnis-T-Zellen (TRMs), die eine direkte Interaktion mit Alloantigenen ermöglichen. Anders als bei anderen Organen erfolgt die Immunaktivierung in der Lunge unmittelbar vor Ort im Transplantat, ohne dass eine Migration in sekundäre lymphatische Organe notwendig ist.

Diese lokale Immunaktivierung stellt eine zentrale Besonderheit der Lunge dar und unterscheidet sich deutlich von der Immunbiologie anderer solider Organe. Sie trägt maßgeblich zu den komplexen immunologischen Herausforderungen bei, die mit einer Lungentransplantation verbunden sind 49 .

## Abstoßung nach Lungentransplantation

Die Transplantatabstoßung ist eine der häufigsten und schwerwiegendsten Komplikationen nach Lungentransplantation. Sie kann akut oder chronisch verlaufen und ist das Ergebnis komplexer zellulärer wie auch humoraler Immunantworten. Zusätzlich spielen nicht alloimmune Faktoren wie IRI oder Infektionen eine Rolle, die die Immunreaktion verstärken und den Verlauf der Abstoßung beeinflussen können. Fortschritte in der Therapie und Prävention haben die Prognose von Lungentransplantierten deutlich verbessert. Dennoch bleibt die Abstoßung eine der Hauptursachen für Transplantatversagen und Mortalität.

### Hyperakute Abstoßung und primäre Graftdysfunktion (PGD)

Die hyperakute Abstoßung tritt meist innerhalb von Minuten bis Stunden nach der Transplantation auf und wird durch präformierte Antikörper des Empfängers gegen HLA- oder Non-HLA-Antigene (z. B. AB0-Antigene) des Spenders ausgelöst 50 51 . Diese präformierten Antikörper aktivieren die Komplementkaskade, was zu einer Nekrose der Endothelzellen, Freilegung der Basalmembran, Aktivierung der Gerinnungskaskade und letztlich zur nekrotischen Gewebedestruktion führt. Klinisch manifestiert sich die hyperakute Abstoßung durch eine rapide Verschlechterung der Oxygenierung und eine refraktäre respiratorische Insuffizienz 52 . Die Therapie der hyperakuten Abstoßung orientiert sich an der Behandlung der akuten humoralen Abstoßung. Durch moderne Screening-Methoden wie Luminex-Assays ist die hyperakute Abstoßung mittlerweile eine äußerst seltene Komplikation geworden, da präformierte Antikörper bereits vor der Transplantation zuverlässig erkannt und im Sinne des virtuellen Crossmatch berücksichtigt werden können.

Die primäre Graftdysfunktion (PGD) ist eine Komplikation, die in den ersten 72 Stunden nach einer Lungentransplantation auftreten kann 53 . Zur Identifikation und Schweregradbeurteilung werden radiologische Befunde (pulmonale Infiltrate im Lungenröntgen) sowie der Oxygenierungsindex (PaO _2_ /FiO _2_ -Ratio) herangezogen. Die Einteilung erfolgt nach einem standardisierten Grading-System (PGD 0–3), wobei Grad 3 die schwerste Form darstellt und die führende Ursache für eine erhöhte Mortalität in der frühen postoperativen Phase ist 54 . Die Pathophysiologie der PGD ist primär auf eine Ischämie-Reperfusions-Verletzung (IRI) zurückzuführen, die zu einem Lungenödem und diffuser alveolärer Schädigung führt 55 . Ein typisches Merkmal ist die endotheliale Dysfunktion, die durch die massive Freisetzung proinflammatorischer Zytokine infolge von Hypoxie und Reoxygenierung ausgelöst wird. Diese Prozesse beeinträchtigen die Integrität des Endothels und erhöhen die Kapillarpermeabilität im Alveolarraum. Während der Reperfusionsphase führen oxidativer Stress und die Expression von Zelladhäsionsmolekülen zu einer Schwellung des Endothels sowie zur Ablösung von der Basalmembran, was das Migrieren von Leukozyten in das Gewebe begünstigt 56 . Die Behandlung der primären Graftdysfunktion besteht aus supportiven Maßnahmen, einschl. restriktivem Flüssigkeitsmanagement, lungenprotektiver Beatmung und ggf. inhalativer Vasodilatatoren (z. B. Stickstoffmonoxid). Bei therapierefraktärer Hypoxämie wird die extrakorporale Membranoxygenierung (ECMO) eingesetzt. Ein frühzeitiger Einsatz kann hierbei prognostisch entscheidend sein 57 .

### Akute zelluläre Abstoßung (ACR)

Die akute zelluläre Abstoßung (ACR) ist die häufigste Form der akuten Abstoßung nach einer Lungentransplantation und stellt eine T-Zell-vermittelte alloimmune Reaktion dar, bei der fremde HLA-Antigene des Transplantats erkannt werden. Sie tritt vor allem innerhalb der ersten 6 Monate nach der Transplantation auf, wobei die Inzidenzrate durch den Einsatz von Tacrolimus und neuen Induktionsstrategien in den letzten 2 Jahrzehnten deutlich gesenkt werden konnte 52 . Sie beträgt heute 10–30%, abhängig von der Art der Immunsuppression 58 . Die meisten Patienten bleiben asymptomatisch und die Diagnose erfolgt häufig durch transbronchiale Biopsien im Rahmen der Nachsorge. Sofern Symptome auftreten, sind diese unspezifisch und umfassen Dyspnoe, Hypoxämie, leichtes Fieber, Husten oder moderate Leukozytose. Bildgebende Verfahren wie Röntgenaufnahmen des Thorax zeigten oft diffuse perihiläre Infiltrate, während CT-Aufnahmen diffuse Konsolidierungen und Infiltrate darstellen können. Die histologische Untersuchung der Biopsie bleibt der Goldstandard zur Diagnosestellung 52 . Histologisch zeigen sich lymphohistiozytäre Infiltrate, die kleine Arteriolen, Venolen und Bronchiolen betreffen können, begleitet von subendothelialen Infiltrationen durch mononukleäre Zellen. Die Einteilung der ACR erfolgt nach dem Schema der International Society for Heart and Lung Transplantation (ISHLT). Dieses Schema unterscheidet 5 Schweregrade der vaskulären Abstoßung. Im vaskulären Bereich wird die Schwere der Entzündung von A0 (keine Abstoßung) bis A4 (schwere Infiltrate mit alveolärer Pneumozytenschädigung, Endotheliitis, nekrotischen Epithelzellen, hyalinen Membranen, Hämorrhagie und Neutrophilen) eingeteilt. Eine parallele Klassifikation der bronchialen Abstoßung erfolgt von B0 (keine Abstoßung) bis B2R (hochgradige Infiltrate mit epithelialen Schäden) 59 . Die Therapie hängt von der Schwere der Abstoßung ab. Geringgradige Befunde (A1, B0–B1R) erfordern häufig keine unmittelbare Intervention. Moderat bis hochgradige Abstoßungen (≥ A2, B2R) werden i. d. R. mit hochdosierten Glukokortikoiden behandelt, die über 3–5 Tage verabreicht werden. Persistierende oder therapierefraktäre Abstoßungen können eine Eskalation der immunsuppressiven Therapie, einschl. der Gabe von Antithymozytenglobulin (ATG) erfordern 52 60 .

Dank verbesserter Behandlungsstrategien ist die Mortalitätsrate durch ACR heute niedrig. Dennoch bleibt die ACR ein signifikanter Risikofaktor für die Entwicklung einer chronischen Lungenallograft-Dysfunktion (CLAD).

### Akute humorale Abstoßung (AMR)

Die akute humorale Abstoßung (AMR) ist eine durch donorspezifische Antikörper (DSA) vermittelte Reaktion gegen HLA- oder Non-HLA-Antigene des Transplantats. Die Pathogenese umfasst sowohl komplementabhängige als auch komplementunabhängige Mechanismen.

Komplementabhängige Prozesse führen über die Bildung des Membranangriffskomplexes (MAC) zu einer direkten Endothelschädigung, die die Basalmembran exponiert und die Gerinnungskaskade aktiviert. Dies resultiert in Thrombosen und nekrotischer Gewebedestruktion. Parallel dazu spielen komplementunabhängige Mechanismen eine Rolle, darunter die Aktivierung von Signalwegen wie PI3K und mTOR. Diese Mechanismen fördern Zellproliferation, Entzündung und Thrombozytenaktivierung. Histologisch zeigt sich eine Infiltration von Neutrophilen, NK-Zellen und Monozyten, begleitet von neutrophiler Margination, Kapillaritis und Arteriitis 61 62 .

Die Diagnose der akuten humoralen Abstoßung basiert auf den Kriterien der International Society for Heart and Lung Transplantation (ISHLT). Diese umfassen die akute Transplantatdysfunktion (klinisch oder subklinisch), den Nachweis zirkulierender donorspezifischer Antikörper (DSA), histopathologische Hinweise auf eine akute Lungenschädigung, subendotheliale C4d-Ablagerungen in alveolären Kapillaren sowie der Ausschluss anderer Ursachen wie Infektionen. Je nach Anzahl und Kombination der erfüllten Kriterien erfolgt die Unterteilung in „definite“, „probable“, und „possible“ AMR 62 . Die Behandlung der AMR stellt aufgrund fehlender randomisierter Daten und zugelassener spezifischer Medikamente eine Herausforderung dar. Die häufig eingesetzten Therapien zielen auf die Reduktion zirkulierender Antikörper ab, da die AMR oft nicht auf konventionelle Immunsuppressiva wie hochdosierte Glukokortikoide anspricht. Zu den angewandten Ansätzen gehören intravenöse Immunglobuline (IVIG), therapeutische Plasmapherese oder Immunadsorption, Rituximab, Proteasomen-Inhibitoren (z. B. Bortezomib oder Carfilzomib) und Eculizumab. Die Wahl der Therapie richtet sich nach Schweregrad, klinischem Verlauf und Ansprechen 61 63 . Trotz intensiver Behandlung ist die Prognose der AMR ungünstig. Die Mortalitätsrate liegt bei 50–70% und viele Patienten entwickeln langfristig eine chronische Lungentransplantatdysfunktion (CLAD) 62 .

### Chronische Lungenallograft-Dysfunktion (CLAD)

Das chronische Transplantatversagen (Chronic Lung Allograft Dysfunction, CLAD) ist die häufigste Ursache für Morbidität und Mortalität nach einer Lungentransplantation. Sie beschreibt eine signifikante und anhaltende Verschlechterung der Lungenfunktion, definiert durch eine Verminderung des forcierten exspiratorischen Volumens in einer Sekunde (FEV _1_ ) um ≥ 20% im Vergleich zum postoperativen Ausgangswert. Diese Verschlechterung tritt in Abwesenheit anderer erklärbarer Ursachen auf, wie z. B. einer akuten Abstoßung oder Infektion 64 . Nach den Richtlinien der International Society for Heart and Lung Transplantation (ISHLT) dient CLAD als Überbegriff für verschiedene Phänotypen. Die beiden Hauptformen sind das Bronchiolitis-obliterans-Syndrom (BOS), das rund 70% der Fälle ausmacht, und das restriktive Allograft-Syndrom (RAS). Darüber hinaus können Mischformen und nicht eindeutig zuzuordnende Phänotypen vorkommen 65 .

Die Differenzierung der Phänotypen ist für die Prognose und das therapeutische Management entscheidend. Das BOS gilt als primär durch inflammatorische Prozesse in den Bronchiolen verursacht und zeigt sich durch eine überwiegend obstruktive Ventilationsstörung (FEV erniedrigt, TLC normal oder erhöht). Radiologisch finden sich in fortgeschrittenen Stadien Mosaikmuster, Bronchiektasen, Air Trapping und Wandverdickungen. Das RAS hingegen entsteht durch fibrotische Umbauvorgänge im Lungenparenchym, was sich in einer restriktiven Ventilationsstörung (TLC und FEV _1_ erniedrigt) und charakteristischen CT-Mustern (Milchglastrübungen, retikuläre Verdichtungen, verdickte Interlobulärsepten) widerspiegelt. Während das BOS oftmals schleichend voranschreitet, kann sich das RAS durch akute Exazerbationen bemerkbar machen 64 66 .

Die Ätiologie der CLAD ist multifaktoriell und wird häufig als Endpunkt unterschiedlicher Schädigungen des Transplantats gesehen. Zu den wichtigsten Risikofaktoren zählen rezidivierende oder schwere Episoden akuter Abstoßung (ACR), lymphozytäre Bronchiolitis (LB), Infektionen (z. B. CMV, Pseudomonas aeruginosa, Aspergillus fumigatus), gastroösophagealer Reflux und PGD. Übergeordnet spielen sowohl alloimmune als auch nicht alloimmune Mechanismen eine Rolle. Es handelt sich um ein komplexes Zusammenwirken von Immunantworten, Entzündung, Fibrose und mechanischen Schäden 66 .

Auf zellulärer Ebene sind verschiedene T-Helfer-Subtypen (v. a. Th1, Th2 und Th17) sowie regulatorische T-Zellen (Tregs) zentral an der Entstehung und dem Verlauf der CLAD beteiligt. Th17-Zellen vermitteln insbesondere die Rekrutierung von Neutrophilen und fördern die Bildung tertiärer Lymphorgane im Transplantat (z. B. induziertes bronchusassoziiertes lymphatisches Gewebe, iBALT). Diese tertiären Lymphorgane beherbergen B-Zellen, die dort zu Gedächtniszellen und Plasmazellen heranreifen und Antikörper produzieren können. Tregs hingegen wirken immunregulatorisch, indem sie durch Zell-Zell-Kontakte und lösliche Mediatoren (IL-10, TGF-β) überschießende Immunantworten bremsen. Eine hohe Tregs-Dichte im Allograft korreliert mit besserer Lungenfunktion, während ein Rückgang dieser Zellen bei fortschreitender CLAD beobachtet wird. Die ACR und LB, die beide T-Zell-vermittelt sind, können den initialen Schaden am Transplantat wesentlich verstärken und so ein chronisches Entzündungsgeschehen befeuern. Treten solche Prozesse wiederholt oder sehr schwer auf, steigt das Risiko, dass das Transplantat langfristig fibrotisch umgebaut wird und sich CLAD manifestiert 67 .

Neben den T-Zell-vermittelten Prozessen gewinnt die humorale Immunantwort zunehmend an Bedeutung. Donorspezifische Antikörper (DSA) gegen HLA- oder Non-HLA-Antigene können das Transplantat erheblich schädigen, indem komplement- und endothelzellabhängige Entzündungskaskaden angestoßen werden. In tertiären Lymphorganen wird die Produktion dieser Antikörper durch eine andauernde Stimulation von B-Zellen aufrechterhalten, was eine permanente Aktivierung der Immunantwort begünstigt. Das AMR wird daher nicht nur als akutes Phänomen betrachtet, sondern trägt auch langfristig zu Strukturschäden im Sinne eines CLAD bei 66 67 .

Die Behandlung der CLAD bleibt eine große Herausforderung, da es keine standardisierten Therapieprotokolle gibt. Beim BOS werden häufig Azithromycin als Erstlinientherapie sowie eine Anpassung der immunsuppressiven Medikation eingesetzt. Ebenso kommen experimentelle Therapien wie die extrakorporale Photopherese (ECP) in Betracht, die möglicherweise sowohl T-Zell- als auch B-Zell-Reaktionen modulieren kann. Für RAS sind bislang keine bewährten Standardtherapien verfügbar, sodass hier vor allem das Risiko-Nutzen-Verhältnis von eskalierender Immunsuppression und verschiedenen experimentellen Ansätzen abgewogen wird. Bei fortgeschrittener CLAD kann letztlich eine Retransplantation die letzte Behandlungsoption darstellen 64 .

## Immunsuppressive Therapie nach Lungentransplantation

Die immunsuppressive Therapie ist eine essenzielle Grundlage der Lungentransplantation. Ihr Hauptziel besteht darin, das transplantierte Organ vor einer Abstoßung durch das Immunsystem des Empfängers zu schützen. Gleichzeitig soll das Risiko für Nebenwirkungen wie opportunistische Infektionen und die Entwicklung von Malignomen minimiert werden. Seit der ersten erfolgreichen Lungentransplantation in den 1960er-Jahren haben sich die Strategien zur Immunsuppression erheblich weiterentwickelt. Ursprünglich wurden Kombinationen aus Azathioprin, Kortikosteroiden und Strahlentherapie eingesetzt, um die Immunantwort des Körpers zu unterdrücken 68 . Heutzutage basieren die Behandlungsprotokolle auf modernen Medikamentenkombinationen, die gezielt in unterschiedliche immunologische Prozesse eingreifen.

Die Prinzipien der Immunsuppression orientieren sich an Erfahrungen aus der Transplantation anderer solider Organe. Die Therapie ist in den ersten Monaten nach der Transplantation intensiv und wird schrittweise reduziert, um langfristig die niedrigste effektive Dosierung zu finden. Dabei werden Substanzen mit unterschiedlichen Wirkmechanismen kombiniert, um immunologische Prozesse, die eine Abstoßung auslösen könnten, effektiv zu blockieren. Eine Überimmunsuppression wird vermieden, da sie das Risiko schwerwiegender Komplikationen wie Infektionen und maligne Erkrankungen erhöht.

Laut der International Society for Heart and Lung Transplantation (ISHLT) umfassen gängige Immunsuppressionsprotokolle eine Induktionstherapie, gefolgt von einer Erhaltungstherapie aus einer Dreifachkombination aus Calcineurininhibitor, einer antiproliferativen Substanz und Kortikosteroiden 69 . Die spezifischen Protokolle variieren jedoch zwischen den Transplantationszentren.

### Induktionstherapie

Die Induktionstherapie beschreibt den gezielten Einsatz potenter immunsuppressiver Wirkstoffe in der unmittelbaren postoperativen Phase, um die initiale starke T-Zell-Antwort auf das transplantierte Organ zu reduzieren. Sie zielt primär darauf ab, T-Lymphozyten zu depletieren und/oder deren Aktivierung und Proliferation zu hemmen. Eine wichtige Zielstruktur ist hierbei der Interleukin-2-Rezeptor (CD25), dessen Blockade die T-Zell-Proliferation unterdrückt und so die Immunantwort moduliert 70 . Zu den häufig verwendeten Wirkstoffen gehören monoklonale Antikörper wie Alemtuzumab oder Basiliximab und polyklonale Antikörper wie Antithymozytenglobulin (ATG). Der Einsatz der Induktionstherapie reduziert die Rate an akuten Abstoßungen und ermöglicht möglicherweise eine Reduktion der Erhaltungsdosis von Calcineurininhibitoren. Nicht alle Transplantationszentren setzen eine Induktionstherapie ein. Nach ISHLT-Registerdaten erhielten im Jahr 2018 über 80% der Lungentransplantationsempfänger eine Form der Induktionstherapie. Dabei wurde Basiliximab bei mehr als 70% der Patienten verwendet, während Alemtuzumab oder ATG seltener eingesetzt wurden 3 71 .

**Basiliximab**

Basiliximab ist ein chimärer monoklonaler Antikörper, der gezielt die Alpha-Untereinheit des Interleukin-2-Rezeptors (IL-2R oder auch CD25) auf der Oberfläche aktivierter T-Zellen bindet. Diese Bindung blockiert spezifisch die Interaktion von Interleukin-2 mit seinem Rezeptor, wodurch die IL-2-vermittelte Proliferation und Differenzierung von T-Zellen effektiv gehemmt wird. Im Gegensatz zu depletierenden Antikörpern führt Basiliximab nicht zur T-Zell-Depletion 72 . Basiliximab wird intraoperativ oder innerhalb von 2 h nach der Transplantation verabreicht, wobei eine 2. Dosis typischerweise am 4. postoperativen Tag gegeben wird. Basiliximab ist gut verträglich und führt im Vergleich zu anderen Antikörpern, wie Alemtuzumab, nicht zu einem Zytokinfreisetzungssyndrom 73 . In seltenen Fällen wurde jedoch über schwere nicht kardiogene Lungenödeme berichtet, insbesondere bei nierentransplantierten Patienten 74 .

**Alemtuzumab**

Alemtuzumab ist ein humanisierter monoklonaler Antikörper, der gegen das CD52-Antigen gerichtet ist, welches auf nahezu allen T- und B-Lymphozyten sowie auf natürlichen Killerzellen (NK-Zellen), Monozyten und Makrophagen exprimiert ist. Die Bindung von Alemtuzumab an CD52 führt zu einer komplementvermittelten und direkten zellulären Zytotoxizität, die eine Depletion dieser Zellen bewirkt. Insbesondere resultiert die Anwendung in einer langanhaltenden T-Zell- und B-Zell-Depletion. Zusätzlich beeinflusst Alemtuzumab die Reifung von Immunzellen und fördert ein tolerogenes Immunmilieu, das zur Verringerung von Abstoßungsreaktionen beiträgt 73 . Alemtuzumab wird als Einzeldosis von 30 mg intravenös oder subkutan zum Zeitpunkt der Organperfusion oder unmittelbar nach der Transplantation verabreicht. Aufgrund des Risikos von Infusionsreaktionen, wie das Zytokinfreisetzungssyndrom, wird eine präemptive Behandlung mit Methylprednisolon, Diphenhydramin und Acetaminophen empfohlen. Neben den akuten Infusionsreaktionen gehören Lymphopenie, opportunistische Infektionen und sekundäre Autoimmunerkrankungen zu den bekannten Nebenwirkungen 75 . Aktuelle Studien zeigten, dass die Verwendung von Alemtuzumab eine verlängerte Freiheit von akuter Abstoßung und eine mögliche Überlebensverbesserung bieten kann, insbesondere im Vergleich zu Patienten ohne Induktionstherapie 76 77 .

**Antithymozytenglobulin (ATG)**

Antithymozytenglobulin (ATG) ist eine polyklonale Antikörperpräparation, die aus Pferden oder Kaninchen gewonnen wird, nachdem diese mit menschlichen Thymozyten immunisiert wurden. ATG enthält Antikörper gegen verschiedene Oberflächenmoleküle von T-Zellen, einschl. Fc-Rezeptoren und anderer Proteine, wodurch zytotoxische T-Zellen funktionell inaktiviert und depletiert werden. Zusätzlich induziert ATG die Apoptose von B-Zellen, moduliert Interaktionen zwischen Leukozyten und Endothelzellen, beeinflusst die Funktion dendritischer Zellen und fördert die Entwicklung regulatorischer T-Zellen sowie natürlicher Killerzellen. ATG wird häufig intraoperativ begonnen, wobei die Dosierung gewichtsabhängig erfolgt. Typischerweise wird rATG 3–5 Tage hintereinander verabreicht. Wie bei Alemtuzumab wird hier ebenso eine Prämedikation mit Glukokortikoiden, Antihistaminika und Antipyretika empfohlen. Nebenwirkungen umfassen infusionsbedingte Reaktionen, Leukopenie, Thrombozytopenie, immunkomplexvermittelte Glomerulonephritis, Serumkrankheit und in seltenen Fällen ein Zytokinfreisetzungssyndrom 73 . Während randomisiert-kontrollierte Studien keinen signifikanten Unterschied in Bezug auf akute Abstoßungen, Bronchiolitis-obliterans-Syndrom oder Überleben im Vergleich zu anderen Wirkstoffen zeigten, deuten retrospektive Daten darauf hin, dass ATG das Risiko von BOS um 20–32% im Vergleich zu keiner Induktionstherapie reduziert 78 79 .

### Erhaltungstherapie

Die Erhaltungstherapie stellt eine lebenslange Behandlung dar, um akute und chronische Abstoßungsreaktionen zu verhindern. Dabei soll das Risiko für Nebenwirkungen wie Nephrotoxizität, Infektionen oder maligne Erkrankungen minimiert werden. Typischerweise besteht die Erhaltungstherapie aus einer Kombination aus einem Calcineurininhibitor (CNI), einem antiproliferativen Wirkstoff und Kortikosteroiden. In der Vergangenheit wurden häufig Ciclosporin und Azathioprin in Kombination mit Prednisolon verwendet, ihr Einsatz hat jedoch in den letzten Jahren deutlich abgenommen. Aktuelle ISHLT-Daten zeigen, dass Tacrolimus, Mycophenolatmofetil (MMF) und Prednisolon heute die am häufigsten eingesetzten Substanzen sind 3 .

**Calcineurininhibitoren (CNIs)**

Calcineurininhibitoren (CNIs) sind eine zentrale Komponente der Erhaltungstherapie nach Lungentransplantation, da sie die Aktivierung und Proliferation von T-Lymphozyten durch Hemmung der Transkription proinflammatorischer Zytokine, insbesondere Interleukin-2, effektiv unterdrücken. Zu den beiden verfügbaren CNIs gehören Ciclosporin und Tacrolimus, wobei letzteres mittlerweile am häufigsten verwendet wird 80 .

Ciclosporin war der erste zugelassene CNI und wurde 1983 von der FDA für die klinische Anwendung zugelassen. Es bindet an Cyclophilin in T-Zellen, wodurch ein Komplex gebildet wird, der die Aktivität von Calcineurin hemmt und somit die Transkription von Interleukin-2 verhindert 81 . Die Nebenwirkungen von Ciclosporin umfassen Nephrotoxizität, Hypertonie, Hyperlipidämie, neurotoxische Effekte wie das reversible posteriore Enzephalopathiesyndrom (PRES), Diabetes, Hirsutismus und Gingivahyperplasie 82 .

Tacrolimus, auch als FK506 bekannt, wurde 1997 zugelassen und ist 10- bis 100-mal potenter als Ciclosporin. Es bindet an FKBP12 in T-Zellen, wodurch ein ähnlicher Mechanismus der Calcineurinhemmung ausgelöst wird. Tacrolimus zeigt ähnliche Nebenwirkungen wie Ciclosporin jedoch mit möglicherweise weniger Hypertonie und Hyperlipidämie, dafür häufiger mit Neurotoxizität und Diabetes 83 .

Beide CNIs werden durch die hepatischen Cytochrom-P450–3A4- und -3A5-Enzyme sowie durch P-Glykoprotein metabolisiert, was zu signifikanten Wechselwirkungen mit CYP-Induktoren (z. B. Rifampicin) und -Inhibitoren (z. B. Makrolide) führt 82 83 . Trotz ihrer Unterschiede zeigen Studien keinen signifikanten Unterschied in der Häufigkeit von akuten Abstoßungen oder Überleben 84 . Neuere Daten deuten jedoch daraufhin, dass Tacrolimus ein geringeres Risiko für BOS aufweist. Laut ISHLT-Daten wurde Tacrolimus bei 83% der Patienten 1 Jahr nach Transplantation und bei 77% 5 Jahre nach Transplantation eingesetzt 84 .

### Antiproliferative Substanzen

Zu den primär eingesetzten antiproliferativen Substanzen in der Lungentransplantation gehören Azathioprin und MMF. Beide Wirkstoffe hemmen gezielt die Proliferation von T- und B-Lymphozyten, indem sie die Purinsynthese beeinträchtigen, die für die Zellteilung dieser Immunzellen essenziell ist 80 .

Azathioprin wird intrazellulär zu 6-Mercaptopurin metabolisiert, das wiederum in Verbindungen umgewandelt wird, die die Synthese von Adenin- und Guanin-Ribonukleotiden hemmen. Dies führt zu einer Reduktion von B- und T-Lymphozyten, einer verminderten Immunglobulinproduktion und einer geringeren Interleukin-2-Sekretion. Nebenwirkungen umfassen unter anderem Leukopenie, Anämie, Thrombozytopenie, Hepatotoxizität und Pankreatitis 85 .

Mycophenolatmofetil wirkt durch die Hemmung der Inosinmonophosphat-Dehydrogenase, wodurch die Proliferation von B- und T-Zellen unterdrückt wird. Zusätzlich kann MMF Apoptose in aktivierten T-Zellen auslösen und die Expression von Adhäsionsmolekülen hemmen, die für die Rekrutierung von Lymphozyten wichtig sind. Nebenwirkungen umfassen Leukopenie, Anämie, Thrombozytopenie sowie gastrointestinale Beschwerden 86 .

Vergleichende Studien zu Azathioprin und MMF zeigen gemischte Ergebnisse. Während eine Studie von Speich et al. einen Vorteil von MMF bei der Verringerung akuter Abstoßungen und der Prävention von BOS aufzeigte, fand eine andere randomisierte Studie von McNeil et al. keine Unterschiede in Abstoßungsraten, BOS und Überleben 87 88 . Jedoch wurde Azathioprin häufiger aufgrund mangelnder therapeutischer Wirkung abgesetzt als MMF. Insgesamt wird MMF aufgrund seiner besseren Verträglichkeit und möglicherweise günstigeren Ergebnisse zunehmend bevorzugt 89 .

### mTOR-Inhibitoren

mTOR-Inhibitoren wie Sirolimus und Everolimus werden zunehmend als ergänzende immunsuppressive Mittel nach Lungentransplantation eingesetzt, insbesondere um die Dosis von CNIs zu reduzieren und die Nierenfunktion zu schützen. Beide Substanzen binden an das intrazelluläre FKBP-12, hemmen jedoch nicht Calcineurin, sondern die mTOR-Signalwege, die für die IL-2-vermittelte Zellzyklusprogression von der G1- in die S-Phase erforderlich sind. Durch diese Hemmung wird die Proliferation von T-Lymphozyten und Fibroblasten reduziert, was auch in der Behandlung und Prävention von CLAD von Interesse ist 80 90 . Die häufigsten Nebenwirkungen umfassen Leukopenie, Thrombozytopenie, Hyperlipidämie, verzögerte Wundheilung und Pneumonitis. Aufgrund des erhöhten Risikos schwerer Wundheilungsstörungen, einschl. bronchialer Anastomoseninsuffizienzen, werden mTOR-Inhibitoren i. d. R. erst ab 3 Monaten nach der Transplantation eingesetzt 73 .

### Kortikosteroide

Kortikosteroide haben seit den Anfängen der soliden Organtransplantation eine zentrale Rolle in der Erhaltungstherapie. Die am häufigsten verwendeten Kortikosteroide in der Lungentransplantation sind Methylprednisolon und Prednisolon. Kortikosteroide wirken durch vielfältige Mechanismen immunsuppressiv, einschl. der Hemmung des NF-κB-Signalwegs, der Verhinderung der T-Zell-Proliferation, der Reduktion der Zytokinproduktion, der Hemmung der Makrophagenaktivierung sowie der Beeinflussung der Lymphozytenmigration. Die langfristige Anwendung von Kortikosteroiden ist jedoch mit zahlreichen Nebenwirkungen verbunden, darunter Hypertonie, Gewichtszunahme, Hyperlipidämie, Diabetes mellitus, Osteoporose, Wundheilungsstörungen und psychiatrischen Veränderungen. Das Ziel ist es daher die niedrigste wirksame Erhaltungsdosis beizubehalten, um eine stabile Lungenfunktion zu gewährleisten und Nebenwirkungen zu minimieren 71 73 .
